# Participatory appraisal for healthcare and welfare management strategies of donkeys (*Equus ascinus*) in Balochistan, Pakistan

**DOI:** 10.3389/fvets.2022.1005079

**Published:** 2022-09-02

**Authors:** Kashif Kamran, Ali Akbar, Mahrukh Naseem, Abdul Samad, Samiullah, Jahangir Khan Achakzai, Zia Ur Rehman, Muhammad Sohail Sajid, Abid Ali

**Affiliations:** ^1^Department of Zoology, University of Balochistan, Quetta, Pakistan; ^2^Department of Microbiology, University of Balochistan, Quetta, Pakistan; ^3^Center for Advanced Studies in Vaccinology and Biotechnology (CASVAB), University of Balochistan, Quetta, Pakistan; ^4^Department of Chemistry, University of Balochistan, Quetta, Pakistan; ^5^Department of Natural and Basic Sciences, University of Turbat, Kech, Balochistan, Pakistan; ^6^Institute of Biochemistry, University of Balochistan, Quetta, Pakistan; ^7^Department of Parasitology, University of Agriculture, Faisalabad, Pakistan; ^8^Department of Epidemiology and Public Health, University of Agriculture, Faisalabad, Pakistan; ^9^Department of Zoology, Abdul Wali Khan University, Mardan, Khyber Pakhtunkhwa, Pakistan

**Keywords:** donkey, participatory epidemiology, *Equus ascinus*, healthcare, donkey owner, Balochistan

## Abstract

In spite of the significant importance of the donkeys (*Equus ascinus*) as draft animal in resource-poor countries like Pakistan, they are equines not receiving the appropriate care. They face challenges including injuries, diseases, lack of basic environment and mismanagement by their owners. The present study aims to provide a brief update on the current status of management of healthcare and the welfare of domestic donkeys using participatory epidemiological tools. These tools can help to provide better strategies for improving their productivity and inclusion in human society. This study was mainly focused only on donkeys and horses, mules and ponies were excluded from the study. We carried out a systematic review of the relevant available published literature and shortlisted 50 articles reporting on the different health related characteristics of donkeys. A comprehensive questionnaire was completed by 191 donkey owners, including nine farriers (all men, average age = 38.24 ± 12.43) over a time span from October 2021 to March 2022. Multivariate Odds Ratios (MORs) and 95% confidence intervals were used to assess the predictions of health management and welfare measures for the surveyed donkeys. The most common observed medical problems in donkey health were hyperlipaemia (28.06%), lameness (16.33%) and dental (20.41%) problems. One-third (34.31%) of the donkeys were underweight. The dull donkey with poor appetite needs a clinical emergency owing to a high risk of developing hyperlipemia, which may be life-threatening. These findings are quite useful for the improvement of healthcare management and the welfare of donkeys.

## Graphical Abstract

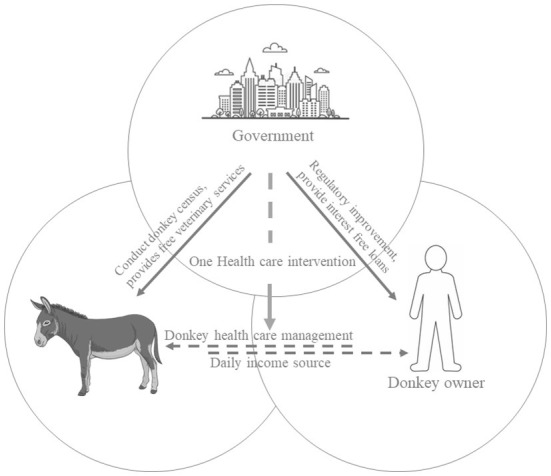

## Introduction

It is estimated that more than half of the world's human population depends on animals as an important source of transportation (1). The latest global donkey population is estimated as 46 million, including 10 million hybrids of donkeys and horses (i.e., mule and hinny). Most of these animals are utilized in low to middle-income countries (2). The majority of the donkey population of the world is concentrated in the Asian continent some countries in Central America and Africa. Pakistan ranked sixteenth largest country based on donkey population in the world (3) (Supplementary material S1). Donkeys are an important species of animals that have made a valuable economic contribution to Pakistani society. For example, donkey riding is considered as an alternative to transport using petrol or diesel in Pakistan due to the rising cost of these fuels. Donkeys enable farmers to access to the neighboring markets. Their use helps to reduce outdoor pollution and the amount of money that can be saved for not using fuels (4). The donkey population in Pakistan has increased from 1.4 million in 1997 to 1.9 million in 2018 (5). The current growth rate of donkeys in Pakistan is increasing at the rate of 2.27% annually. In Balochistan, there are 47,000 working donkeys which provide an important alternative to transportation to underprivileged communities (6). Donkey-owning communities play key role in the animal welfare concerns of the donkeys (7). Government has the most of financial resources therefore its role is crucial in maintaining the health of donkey and their owners through ‘One Health' approach. This is shown in the Graphical Abstract.

Donkeys are economically important domestic animals in rural, peri-urban, as well as urban areas of developing countries like Pakistan. For example, the lack of proper metaled roads in rural areas makes donkeys the most valuable animal for farmers. Compared to expensive vehicles, the donkey carriage is an easy ride which involves only a single person (8). The donkey (Perissodactyla: Equidae) is a unique breed of animal derived from the African wild donkey (*Equus africanus asinus*) (9). Donkey carts play an important role in the rural communities and hilly areas where these are used daily for the transport of goods, timber, water, people, etc. Donkeys work under extreme weather conditions i.e., severe heat or cold and humidity conditions in urban areas and also face various hazards such as heavy traffic, noise, population and debris (10).

For example, working donkeys under deficient nutrition and improper handling lead to severe fatigue to them. They are often overloaded and used for long hours in thermal stress conditions and they face frequent dehydration and high prevalence of lameness (11, 12). Heat stress also lowers the natural immunity making majority of animals more vulnerable to certain diseases (13). However, little effort has been made to quantify and dissolve these issues (14). The skin of donkey is used in the manufacture of traditional Chinese medicines called e'jiao and illegal export of donkey skin from Pakistan to China is also very common (15, 16). As modern means of transport are not readily available in rural areas of Pakistan and therefore the use of donkeys is the first choice in these areas. The purchase and caring of donkeys are quite easy and straight forward. They can be trained and handled easily due to their relatively smaller size. They have a highly efficient digestive system and able to quench their thirst. These advantages make them a popular choice among the resource-poor farmers (16). Donkeys commonly suffer from colic, hyperlipidemia, dental and parasitic diseases (17). In Pakistan, only a few studies have been reported that focus on donkey management and health care (7, 8, 18–20).

Few examples of zoonotic diseases are incorporated in the given paragraph. Lack of medical knowledge, general attitudes and understanding of the factors that affect the risk of zoonotic diseases such as Equine piroplasmosis (21) Equine granulocytic anaplasmosis (22). These diseases are transmitted by ticks in donkeys and prevails in our societies. These factors are also important for the health of humans, livestock and wildlife because they can be a victim of zoonotic diseases transmitted from donkey (22).

The raising of donkeys involves problems like overfeeding, low weight, lack of exercise and poor social interaction. Most equine studies are performed on horses (*Equus caballus*) (23) and a little information is available about the potential zoonotic diseases of donkeys and their possible transmission to their owners. This necessitates to plan an active surveillance study to obtain some reliable data on the donkey owners in Balochistan using participatory epidemiological tools. We expect that the results of our investigation will play an important role in (a) risk-assessment of the zoonotic diseases and (b) identifying preventive management, health care and welfare issues for donkeys of Balochistan.

## Materials and methods

### Study area

**Figure 1:**
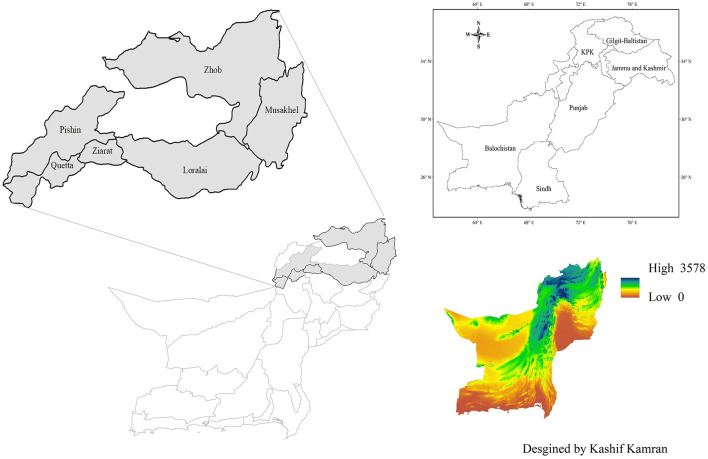
Map of the selected districts of Balochistan province. (Left) The gray area represents the selected districts. (Right) The altitude map of Balochistan [source: Author mapped using the data retrieved from ArcGIS^®^ ArcMap software by Esri (ESRI, CA, USA), accessed on 15 September 2021].

This study was conducted in six districts of Balochistan i.e., Quetta, Pishin, Ziarat, Loralai, Musakhel and Zhob (Figure 1). The criteria for selection of these study districts were (a) the density of donkey population and (b) the highest number of donkey owners in each district (Supplementary material S2). These districts cover an area of about 41,520 km^2^ and include almost half of the donkey's population in Balochistan. The Quetta district (30.2° N and 67° E) is a part of the Northern irrigated plains having a cold and semi-arid climate, which is characterized by its moderate temperatures during summers and low temperatures in winter with little rain. The maximum average temperature in summer and winter is 20°C and 10°C respectively. Mean monthly rainfall in summer and winter is 10.82 mm and 81.63 mm, respectively. Pishin district (30.58° N, 67.01° E) has a maximum temperature of 21°C in summer and a minimum 12°C in winter. The average monthly rainfall is 6 mm during summer and 79 mm during winter. Ziarat district (30.39° N, 67.71° E) has a continental climate, with an average maximum temperature of 20°C in summer and minimum of 10°C in winter. The average monthly rainfall is 14 mm in summer and 44 mm in winter. Loralai district (30.38° N, 68.59° E) has an average maximum temperature of 34 °C in summer and minimum of 25 °C in winter. The average monthly rainfall is 13 mm in summer and 22 mm in winter. Musa Khel district (32.63° N, 71.74° E) has maximum temperature of 22°C in summer and a minimum of 14°C in winter. The average monthly rainfall is 52 mm. Zhob district (31.34° N, 69.46° E) is located in the south of Quetta district and is a part of the western dry mountains having a semi-arid and hot climate. The maximum mean temperature in Zhob district is 22°C in summer and 14°C in winter. The monthly mean rainfall is 53 mm in summer and 49 mm in winter (climate-data.org).

### Database search

**Figure 2:**
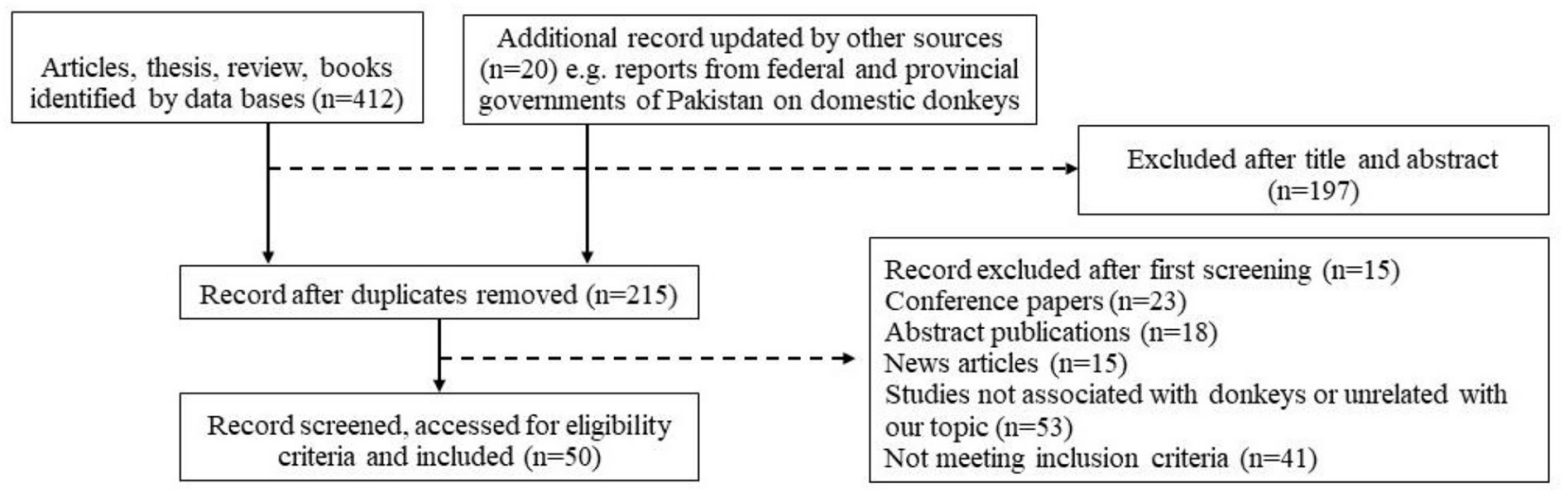
Flow chart representing the selection of studies for inclusion criteria for the systematic review of donkeys and their welfare and healthcare management strategies.

We carried out a systematic review of the relevant available published literature from Web of Science and Scopus till June 30, 2021. We used the following keywords: knowledge, attitude, practices, donkey, donkey owners, donkey diet, body scoring, transport, slaughter, shelter, welfare, healthcare, management and human-animal relationship for our search. From initial 60% abstracts (215 randomly selected from a total of 412), we shortlisted 50 complete articles, short communications, case reports and survey studies, reviews for abstracts and reporting on the given characteristics of donkeys (Figure 2). However, conference papers were not included owing to lack of detailed data.

### Participants and sampling procedure

The study adopted a methodological research design that includes quantitative and qualitative approaches of the participatory rural appraisal technique aimed to quantifying the key variables of the study. A list of donkey owners was compiled with the help of local administrative heads, community groups, management committees and donkey farm managers. Donkey owners were selected (using a systematic random sampling method) from location such as markets, watering points and government veterinary hospitals. The donkeys included in his study did not belong to any particular farm, rather they were donkeys owned by individuals and they are tied inside / outside the house of the owner. The selected respondents were allowed to participate through semi-structured interviews after their formal consent for participation in the surveillance. Participants were informed about the purpose of the study. They were also ensured this data would be utilized only in a publication where their names will not be disclosed to avoid them from any conflict with government officials. The respondents were given an option to withdraw from the study at any stage of the surveillance. Prior to the interviews, written consent was obtained from all the respondents. Their names and addresses were kept anonymous in a password-protected database. Hence, the owners participated with complete information and on purely voluntary basis. The interview was conducted at the owner's house or at any other location as per convenience of the respondents. The interview lasted an average of 90 min (range: 60–120 min). This was necessary because the owner left the house early in the morning and returned late in the evening. All persons interviewed were men.

The basic criteria for involving donkey owners in the study included the following three parameters: (a) The participants had at least 3 years of donkey care experience, (b) The age of the participant was not <18 and (c) Only fully vaccinated against COVID-19 donkey owners were included. A five-page booklet consisting of 56 questions and entitled “Healthcare Management and Welfare of Domestic Donkeys” was initially prepared in English and translated into Pashto. Space for additional written information (open-ended questions) was also given in the questionnaire. The questionnaire is included as Supplementary material S3.

Questionnaires were administered by trained Bachelor of Science (BS) students of the Zoology Department as enumerators who could frequently communicate in the Pashto language. Before donkey owners were interviewed, a joint training session for 1 day was conducted which enabled them to perform and note down the answers contained in the questionnaire.

All questions were asked and answered in Pashto (the most widely spoken language in that community) and later on the response to the questions were translated into English before entry of relevant information into the database. The questionnaire was pretested and certain changes were made as per the need in the questionnaire before its final implementation. The data collected was mainly focused on the following three parameters (a) knowledge about diseases (b) personal protection measures and (c) donkey management methods as described by Stafford (24).

The final comprehensive questionnaire was divided into four sections. Section one Introduction contained questions on the socio-demographic background of the donkey owners (such as age, sex, marital status, urbanity, education, animal ownership and monthly income). The remaining sections of the questionnaire was related to knowledge, attitudes and practices regarding preventive health management and welfare of donkeys. The questionnaire was first cross-examined by three veterinarians and then by traditional knowledge holders. The size of the sample was small owing to lack of complete and up-to-date information about donkey farms provided by the veterinary officers and government officials. It is further certified that no human or animal was harmed during the present study. Respondents who completed the questionnaire were further given 1-week training to improve the health management of their donkeys. Acaricides were also provided to the owners having infested donkeys as a special incentive upon completion of the study.

### Data collection for donkeys

In June 2021, the relevant information including breed, sex, age and animal origin was taken from individual donkey owners. Most animals were brought either from neighboring countries or raised by owners in their farms. The donkeys identified in our study districts were of Shinghari and Sparrow breed. Body conditions of these donkeys were observed for 15 min during daytime for a period of 2 weeks.

### Statistical analyses

A few questions about knowledge, attitude and practices (KAP) were reviewed in the three-point Likert scale format with 'yes,' 'maybe' and 'no' answers. The remaining questions were recorded on a binary results variable based on a yes / no scale (i.e., 1 was for the respondents who had “sufficient knowledge” and 0 was for the respondents who had “insufficient knowledge”). All data were collected in the form of questionnaires and transferred into an Excel file (Microsoft Excel 365^®^). Data analyses included three different sets. In the first set, we developed a frequency table to describe the descriptive variables of the study for socio-demographic characteristics, knowledge, attitudes and practices of the participants. The student *t*-test was applied to the second set for a single sample to determine if there were significant differences between these parameters using IBM SPSS for Windows^®^ (version 19.0, IBM Corp). For the third set, the odd ratio (ORs) for each of the observed variables was calculated using WinEpi-info^®^ (https://www.cdc.gov/epiinfo). The reliability and validity of the results were assessed using the Hosmer-Lemeshow test for the goodness of fitness. The variables associated with the outcome from odds ratios were used to make a population model for housing and management of donkeys. The global donkey population map and the study area were created using ArcGIS software.

## Results

### Systematic review

In the literature review, 412 scientific articles, books were retrieved, of which only 12.12% (50 publications) were found to be directly related to the topic of donkeys, their welfare and healthcare management. A brief summary of systematic review is also given in the abstract i.e., we carried out a systematic review of the relevant available published literature and shortlisted 50 articles reporting on the different health related characteristics of donkeys.

### Profile of the respondents

The socio-demographic pattern of donkey owners and the characteristics of their animals are described in Table 1. A total of 196 donkey owners including nine farriers were included in this study. The proportion of the participants from Quetta district was slightly higher (*n* = 74, 37.76%) other districts while for remaining districts the proportions are as under Pishin (*n* = 60, 30.61%), Zhob (*n* = 17, 8.67%), Musakhel (*n* = 21, 10.71%), Ziarat (*n* = 11, 5.67%), Loralai (*n* = 13, 6.63%). Most owners (*n* = 144, 73.465%, *p* > 0.25) were from urban areas. The majority of respondents (*n* = 91, 46.43%, *p* < 0.009) have their aged around 37. The average age of the donkeys was 8.10 ± 3.48 years. The majority of the donkeys were males (*n* = 213, 93.42%). The most common household size was six members. All respondents were males and the majority of the respondents (*n* = 159, 81.12%) were married. About 62.24% (*n* = 122) of the participants had no formal education while 34.14% (*n* = 67) had completed their primary school education. The factor of “experience in the livestock sector” was not found significantly associated with the collected data (*n* = 54, 62.06%, *p* < 0.27). Majority of respondents (*n* = 175, 89.29%, *p* < 0.29) had their own donkeys. Most respondents (*n* = 141, 71.94%, *p* < 0.28) earned <$200 and they have raised donkeys for more than 10 years (*n* = 121, 61.73%, *p*> 0.04). Most donkeys (*n* = 168, 85.71%) were used for transport purposes and very few were kept as pets in rural Balochistan. One respondent specifically said:

**Table 1:** Sociodemographic breakdown of survey respondents.

**Variable**	**Parameters**	**Frequency**	**Percentage**	***p*** **-value**
Age of the respondent	18–30 years	47	23.98	0.009
	31–40 years	91	46.43	
	Above 40 years	58	29.59	
Urbanicity of donkey owner	Urban	144	73.46	0.25
	Rural	52	25.87	
Marital status	Single	28	14.29	0.47
	Married	159	81.12	
	Divorced	6	3.06	
	Other/Prefer not to answer	3	1.53	
Educational status	College 2 years	0	0	0.27
	Secondary education	7	3.57	
	Primary education	67	34.18	
	Never attended school^1^	122	62.24	
Donkey ownership	Yes	175	89.29	0.49
	No	21	10.71	
Donkey care experience	3–5 years	32	16.33	0.04
	6–10 years	43	21.94	
	More than 10 years	121	61.73	
Average monthly income (US $1 = PKR 178)	<$250	141	71.94	0.28
	$250–300	32	16.33	
	>$300	23	11.73	

1 The respondents under “Never attended school” were considered “Illiterate,” while the rest were considered “Literate” in the analyses.

“The income coming from donkey is used to meet our daily living expenses. Our children also use them for rides. We also provide free rides to other people during our work. Local bodies do not work on regular improvement of road paving. Donkey cart tires often break down prematurely due to poor road conditions in rural areas.” When asked the question “*why are donkeys used for earning by males only*,” one of the replies was as under:

“Donkeys are mostly used as a source of income by males, while it is used by females to bring animal fodder to home or shifting of luggage from one to another house. When we bring our donkeys home in the evening, our wives clean them and serve them with food and water”

### Knowledge-based questionnaire

**Figure 3:**
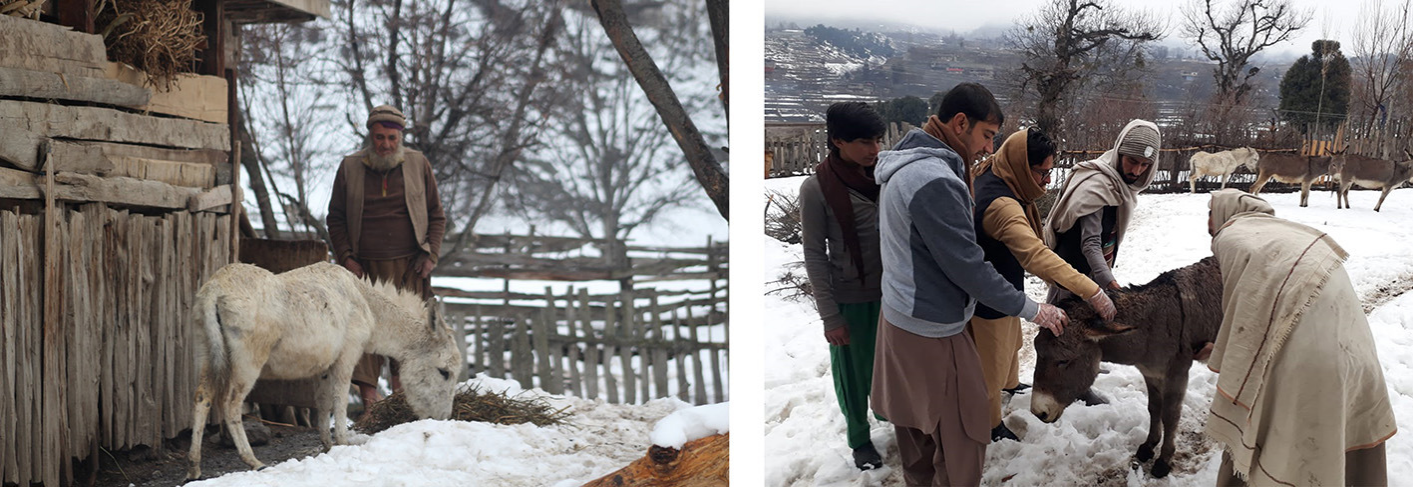
(Left) The most observed lesions – back sore/bruises on the donkey: at the survey site, (Right) Donkeys for sale in the market; note that there is lack of shelter, water or feed (Photo credit: Kashif Kamran). Photo permission for inclusion was obtained from the people shown in the picture.

The results for “knowledge of donkey owners” are given in Table 2. The answer to the question “*Do you know about zoonotic diseases*?” was given as an unexpected “no” (*n* = 160, 81.63%, *p* > 0.09). The majority of donkeys were seen in markets and houses without any shelter (*n* = 165, 84.18%, *p* > 0.25, Figure 3). Most respondents (*n* = 129, 65.82%, *p* < 0.00) were unaware of ectoparasitic treatments. Almost half (*n* = 100, 51.02%, *p* < 0.00) of the respondents have reported that tick infestations do cause the animals to lose weight. Half of the respondents (n = 118, 60.20%, *p* < 0.03) usually gave first aid if the donkey gets too sick to travel during transportation and do not force the animal to continue the journey (*n* = 150, 76.53%, *p* > 0.05). It is a matter of great concern that no surveys so far have been conducted by the government to assess the health status of donkeys (*n* = 180, 91.84%, *p* > 0.54). There is also a lack of awareness about diseases among donkey owners due to the communication gaps i.e., mainly language barrier between veterinarians and donkey owners (*n* = 152, 77.55%, *p* < 0.05). A list of common health problems in donkeys is shown in Table 3. The majority of them reported (63%) a single health problem, while 25% reported two health problems and only 2.5% reported three health problems in the donkey. Hyperlipaemia (*n* =58, 28.06%, *p* < 0.001) was the most frequent disease followed by dental diseases (*n* =40, 20.24%, *p* < 0.001) and lameness (*n* = 32, 16.33%, *p* < 0.001). No treatment for dental diseases was carried out in the last year. The respondents (*n* = 115, 58.67%, *p* > 0.05) mentioned that their donkeys were not vaccinated against influenza and tetanus. One of the respondents commented on vaccination as under:

**Table 2:** Knowledge of donkey owners.

**Survey questions**	**Response**	**Frequencies**	**Percentage**	***p-*** **value**
Are you aware of zoonotic disease?	Yes	36	18.37	0.49
	No	160	81.63	
Knowledge about the external parasite treatment (ticks, lice, or fleas)	Yes	31	15.82	0.00
	No	129	65.82	
	Never examined	36	18.37	
Availability of shelter for donkeys in market/house	Yes	31	15.82	0.25
	No	165	84.18	
Weight reduction in donkey due to ticks	Yes	100	51.02	0.00
	No	96	48.98	
Availability of first aid to injured donkey	Yes	118	60.20	0.03
	No	78	39.80	
Provision of rest for sick donkey	Yes	150	76.53	0.05
	No	46	23.47	
Knowledge of any acarological and parasitological survey by the government	Yes	16	8.16	0.54
	No	180	91.84	
Knowledge of any treatment centers	Yes	135	68.88	0.03
	No	61	31.12	
Medical and technical support by government	Yes	64	32.65	0.33
	No	132	67.35	
Language and the ethnic barrier issues faced by veterinary officers	Present	152	77.55	0.05
	Absent	44	22.45	
Availability of licensed veterinary vaccine for influenza and tetanus	Yes	18	9.18	0.05
	No	115	58.67	
	Don't know	63	32.14	
Health problems in donkeys (*n =* 102)	Ocular	16	8.16	0.001
	Wound	18	9.18	
	Gastric (colic)	13	6.63	
	Lameness	32	16.33	
	Dermal (skin or hair)	22	11.22	
	Hyperlipaemia	55	28.06	
	Dental diseases	40	20.41	

^1^No-response was not considered.

**Table 3:** Attitude of donkey owners.

**Survey questions**	**Response**	**Frequencies**	**Percentage**	***p*** **-value**
Donkey (s) kept with other animals	No	123	62.76	0.05
	With one animal	48	24.49	
	With more than one animal	25	12.76	
Adopted personal protective measures while in contact with other livestock animals (e.g., sheep, cattle, goats, etc.)	Yes	20	10.20	0.35
	No	173	88.27	
	Sometimes	3	1.53	
Participated in the free training conducted by Animal Husbandry Department	Yes	7	3.57	0.54
	No	189	96.43	
Details of feeding methods	Field grazing	64	32.65	0.003
	Hand grazing	132	67.35	
Feeding frequency in a day	Any one time	12	6.12	0.17
	Two-times	135	68.88	
	Three-times	49	25.00	
Cleaning frequency of donkey	Daily	176	89.80	0.17
	Weekly	11	5.61	
	Monthly	8	4.08	
Means used for drinking water	Automatic drinker	4	2.04	0.61
	Bucket	192	97.96	
Consulted a veterinarian for the treatment of infested animals	Yes	78	39.80	0.00
	No	118	60.20	
Whipping frequency per day	One time	18	9.18	0.32
	Multiple times	9	4.59	
	Not using	169	86.22	
Awareness about microchip usage	Yes	185	94.39	0.54
	No	8	4.08	
	Do not know	3	1.53	

“We have no information about the donkey's vaccine and no government official has ever informed us about its availability. We do want our animals to be free of diseases, but for that, we need government help. Although, human vaccine awareness campaigns are conducted throughout the year but there is also a need for animal vaccine awareness campaigns.”

### Attitude based questionnaire

As shown in Table 3, donkeys were mostly kept separately from other animals. Most respondents (*n* = 172, 88.27%, *p* > 0.05) were not using personal protective measures during contact with other livestock animals. Animal welfare and health-related training were not conducted by the government (*n* = 189, 96.43%, *p* > 0.54). Majority of participants (*n* = 118, 60.20%, *p* < 0.00) stated that they were not seeking any medical attention for infested donkeys because of its high cost. The donkey was fed at least twice and every effort was made to keep it clean daily (*n* = 176, 89.80%, *p* < 0.00). Additionally, the respondents stated that all the donkeys were provided with drinking water daily using 2.04% automatic drinkers and 97.96% buckets. There were only 67.34% (*n* = 132/196) of donkeys had access to clean drinking water. Most of the respondents (*n* = 169, 86.22%, *p* > 0.32) were not using whips on donkeys during their working time. One of the participants gave the following reason for not using a whip.

“I prefer not to whip the donkey to avoid any possible injuries. Further animals do have the same feeling of pain as humans. I also persuaded other people not to use the whip. The continued use of whip makes the donkey slow and disobedient to its owner.” As far as the availability and use of microchips are concerned, the majority of the respondents (*n* = 185, 94.39%, *p* > 0.54) stated that the donkeys arrived without a microchip.

### Practice based questionnaire

Data collected about the practice of the donkey owners toward their donkeys were presented in Table 4. Nearly three-quarters of respondents (*n* = 168, 85.71%, *p* > 0.25) stated that they could not afford acaricides due to financial constraints and preferred to use traditional medicines. Only 5.74% of donkey owners reported tick bite prophylaxis experience in the past 2 years.

**Table 4:** Practice of surveyed donkey owners.

**Survey questions**	**Response**	**Frequencies**	**Percentage**	***p-*** **value**
Use of acaricides against tick infestation	Yes	28	14.29	0.25
	No	168	85.71	
Period of farriery services^1^	1–2 weeks	29	14.80	0.00
	2–3 weeks	116	59.18	
	Seasonal	51	26.02	
Reason for acquiring farrier services	Good relation with a farrier	142	72.45	0.009
	Farrier's location	19	9.69	
	Cost and skill	35	17.86	
Killing of donkeys without any reason	Yes	0	0.00	0.63
	No	196	100.00	
Support by local donkey welfare society	Yes	23	11.73	0.33
	No	173	88.27	
Nature of load carried by donkeys	Waste disposal	21	10.71	0.11
	Building materials (e.g., bricks and cement making)	129	65.82	
	Agriculture produce	31	15.82	
	Transportation of firewood and water	15	7.65	
Daily weight carried by a donkey^1^	<100 Kg	14	7.14	0.00
	>100 Kg	39	19.90	
	>150 Kg	54	27.55	
	>200 Kg	89	45.41	
Daily working hours of donkey^2^	<5	21	10.71	0.47
	5	23	11.73	
	> 6	152	77.55	

1 For an average donkey with a weight of 160 kg, the normal load carried is 50 kg (25).

2 Donkeys are usually allowed to work 6 days per week with one full day rest and can work up to 6 to 9 h per day (26).

**Figure 4:**
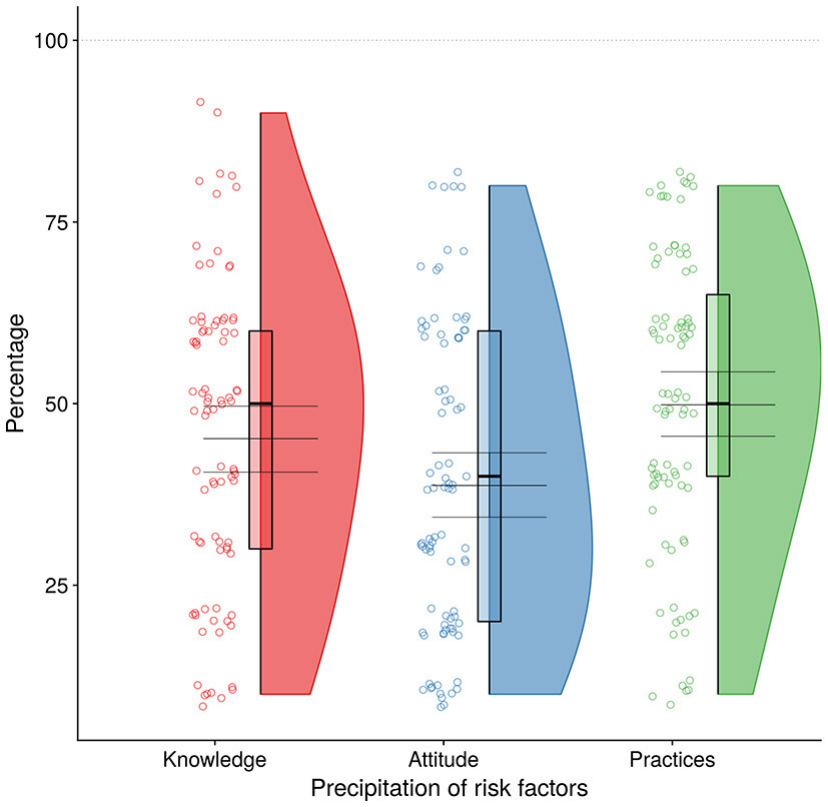
Precipitation of risk factors i.e., knowledge, attitudes and practices.

Regarding the farriery service, most of the respondents (*n* = 116, 59.18%, *p* < 0.00) acquired farriery services every 2 to 3 weeks and only 26% were adjusted this shoeing schedule seasonally. The majority of the respondents (*n* = 76, 87.35%, *p* < 0.009) preferred farrier services from the same farrier owing to a good relationship between them. The average cost for shoeing was 250 Pakistani rupees per hoof. No donkey owner supported the slaughter of donkeys for human consumption on their religious grounds. In terms of load carried by a donkey, construction materials account for 65.82% followed by agricultural products 15.82%, firewood and water transportation 10.715.75% and waste disposal 7.65%. Only 89 donkeys (45.41%, *p* < 0.00) carried above 200 kg. About 77.55% of donkey owners reported the working hours of the donkey exceeded 6 h. Figure 4 gives us a comparison of knowledge, attitudes and practices.

### Housing and animal management

The results for the “predictionary model for donkey health management” are given in Table 5. The Hosmer-Lemeshow test was not applicable for most of the variables. Further, a multivariable model could not be established because the predictor values for each model were higher than 1.00 at a 95% confidence interval. Donkeys were reared mostly on the grassy floors as compared to the cemented ones (OR = 5.04, Cl = 2.11–12.00). Most of the assessed donkeys showed normal body condition with the result of adequate nutrition (OR = 1.97, CI = 0.95–4.0). The houses with a clean sanitation system were statistically significant (OR = 5.18, Cl = 2.21–12.13). In bivariate analysis, it was found (OR = 1.83, CI = 0.88–3.76), which shows a tendency of the owner toward taking their animals to veterinarians only in case of severe illness. Three donkeys died (OR = 6.06, CI = 0.57–63.67) during our study due to illness. However, both of these variables did not show statistical significance (*p* > 0.05). The survival of donkeys is essential to their owners and in this regard, one donkey owner expressed his feelings of grief and loss regarding the death of his donkey:

**Table 5:** Predictionary model for donkey health management.

**Variable/Risk factors**	**Level**	**Positive (%)**	**Negative (%)**	**OR (CI)^a^, ^b^**	***p*** **-value**
Nature of housing floor	Grass	121	9	1 5.04 (2.11–12.00)	0.00
	Cemented	48	18		
Regular cleaning of floor	Yes	143	25	1 3.70 (1.55–8.82)	0.00
	No	17	11		
Good feeding (corelated with body condition score)	Yes	112	21	1	0.03
	No	46	17	1.97 (0.95–4.0)	
Working more than 8 h	Yes	132	11	1	0.00
	No	37	16	5.18 (2.21–12.13)	
Regular checkup by veterinarians	Yes	86	15	1	0.14
	No^e^	72	23	1.83 (0.88–3.76)	
Donkey survival i.e., alive / dead during the study^c^	Alive	182	10	1	0.20
	Dead	3	1	6.06 (0.57–63.67)	
Body condition score of donkeys^d^	Underweight	37	5	1 4.25 (2.75–20.71)	0.00
	Normal	121	7		
	Overweight	15	11		

a Model was designed following the guidelines within the framework of the Pakistan Agriculture Research Council.

b OR, Odds Ratio or Risk Ratio; Cl, lower and upper 95% confidence interval.

c Total number of donkeys that died during the study period (October 2021 to March 2022).

d Accurate body condition scoring is a hands-on process for feeling the amount of muscle and fat that are covering the donkey's bones. Careful assessment of all areas was made and combine to obtained body score.

e It includes donkey owners who treat their animals with home remedies.

“If you raise chickens, then you have to feed them twice a day and your earnings from them will be after 1 month. On the other hand, a donkey is capable of meeting your daily expenses. However, you lose your source of income as soon as the donkey dies. Therefore, I always advise my children that if they want to make a living from donkeys, they should purchase two donkeys. Use one donkey and rent the other one.”

**Figure 5:**
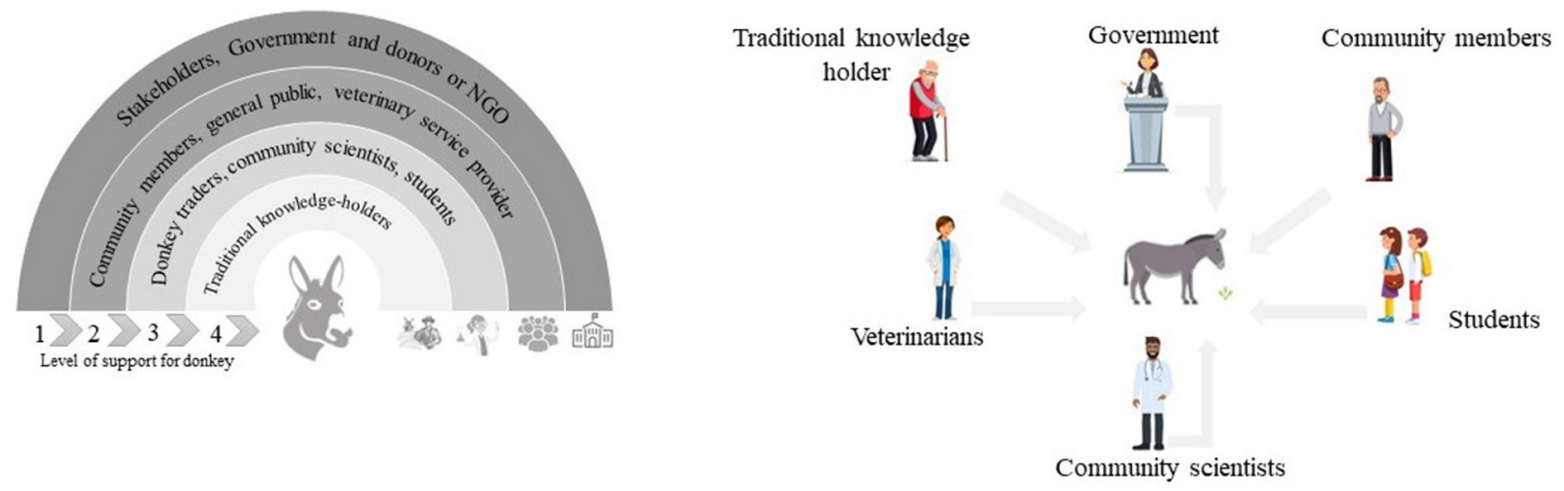
Conceptual models of various characters and working groups for healthcare management and welfare of donkeys. Left: Donkey assisted by collaborating in the practice model shows work overlapping of different communities. The center of the diagram depicts the donkey - the circle begins with the leading role of the government and NGOs in donkey welfare (such as providing legal assistance to improve donkey carriers and chart equipment, adjusting the price for the sale of the individual donkey, traffic police guidelines to run the donkey carts along with vehicles and provision of effective veterinary services) - while individuals who have their role in the co-operative animal project are increasing their participation by moving toward the center. Right: The collective model focuses on mutual engagement, centralized decision-making and collaborative approach to improve donkey policy, develop the relevant manuals, protocols adoption, etc. Each donkey owner treats his donkey from a different point of view such as theoretical and perspective approaches.

Based on this predictionary model, two conceptual models are proposed (Figure 5).

## Discussion

This study represents a significant health care management and welfare problem in cart pulling donkeys. To the best of our knowledge, this study is the first socio-economic analysis of donkey owners which focuses on their knowledge, attitude and practices in Balochistan. In the rural and urban settings donkeys are usually raised for economical purposes and a proficient parasitic control program is not readily available (27). The average age of the donkey owners was 37.19 ± 11.32 years, which was around 20 years higher than those of the owners of donkey herds in China (28). The relative young age of the owners indicates a further possible increase in the population of donkeys in Pakistan in the near future. The majority of the owners had no formal education and this factor might have affected the healthcare management and welfare of the donkeys. The mean ages of the donkeys were 8.10 ± 3.48 years and are at par for donkeys reported in Ethiopia (29). Donkey owners leave their donkeys in other populated urban areas, where they can roam freely even in ill or seriously injured conditions. Some countries such as the United Kingdom have developed sanctuaries for unwanted donkeys (30). It has also been observed that younger donkeys carry extra loads and have been used in donkey carts (18). There were more male donkeys (93.42%) as compared to females which might be due to the fact that most donkey owners prefer male donkeys for load-related tasks based on their perceived strength (31). A higher proportion of male donkeys in China (32), Nigeria (33) and Portugal has been reported.

When donkey owners were asked about zoonotic diseases and external parasites among donkeys, their most apparent answer was in ‘NO.' The main reason for this answer was the lack of awareness among donkey owners. The donkey owners are mostly ignorant of the healthcare management techniques and welfare requirements of donkeys. Further, they were also found untrained and unfamiliar with these requirements. Due to these reasons, it was impossible for them to improve the management of their donkeys. Ignorance is linked to scarcity, illiteracy and lack of knowledge which makes it difficult to achieve the goals of modern agriculture and livestock trends (4). The health status of the donkeys under investigation might have been affected due to the lack of awareness campaigns by the government. The study also indicated that the majority of respondents could not get immediate veterinary attention or treatment for their donkeys because they could not afford the cost of medicines. It happens because donkey owners are unable to properly meet the basic needs of their families and rarely have the ability or desire to solve their animal health issues in a meaningful way (34). The effective use of working animals depends on their management and husbandry (35). These animals are essential for the livelihoods of their owners and it require an adequate support by the state without affecting the health and mental state of the animals (36).

During this course of the study, donkey owners revealed that they got a farriery service for their donkeys after 3 weeks and found no apparent risk. However, these statements contradict our findings where the condition of the hooves appears to be a concern for the welfare of donkeys with neglected symptoms such as overgrowth and/or incorrect trimming with too much variation (37). One of the major benefits of questionnaire-based surveys is the ability to review the routine behavior of the individual respondent. People tend to present a favorable picture of them in response to the questionnaire instead of giving actual facts. This phenomenon is called as a “socially desired response” and this behavior can develop outliers in the survey results by creating a false relationship between the variables (38).

The donkey owners admitted that they do not want to sell their donkeys to the slaughterhouse. However, slaughtering of donkeys is common practice in different region of the world including Kenya (39) and Brazil (40).

The weight of load carried by donkey depends upon the nature of the load i.e., the volume of agricultural waste disposal is large with less weight as compared to construction material. For example, donkeys used for transporting construction loads carry more weight than donkeys used for carrying agricultural loads (41). The type of load carried by a donkey has a unique effect on its health (42). For example, donkeys used to transport brick are 2.5 times more likely to have moderate to deep skin lesions (26). It was reported that in practice, over long distance journey, the loads were much higher than the standard 50 kg/160 kg (25). Globally, overloading is one of the primary welfare concerns for working donkeys (41). Increased weight carrying can have negative effects on the health of donkeys and which can lead to lameness (43). The weight of mounted loads found in our research is also similar to previous investigations from Pakistan (41), Ethiopia (44) and India (45).

Most owners did not fully give due importance to shelters in markets and houses in severe/bad weather conditions. Wooden shelters and iron roof stables were not used for donkeys, which creates physical and mental stress in the early hours of the day in winter (46). It was satisfactory that donkey owners did serve water to their donkeys from time to time. Donkeys have physical characteristics, which allow them to manage their dehydration temporarily and can quickly rehydrate (47).

The ectoparasites such as lices, ticks and mites inhabit the host's skin and depend on their host for their sustenance, maturation and multiplication. Among these, ticks are the most prevalent in various regions of Pakistan (48, 49) probably due to the favorable climatic conditions. It is spreading due to lack of awareness of donkey owners, inadequate veterinary services and indiscriminate use of acaricides (50). Donkeys are hosts of different tick species that commonly bite humans and domestic animals (51, 52). These ticks could act as amplifiers or reservoirs of other zoonotic tick-borne pathogens such as *Anaplasma phagocytophilum, Coxiella burnetii, Borrelia* spp., *Leishmania* spp. and *Rickettsia* spp (51, 53). Ectoparasites such as diptera causing myiasis, lice and scabies that affect the skin of the donkeys (54) and skin lesions due to them can be seen particularly on the limbs of the donkeys (55). For example, in Figure 3, the picture on the left depicts the most common lesions such as back sore or bruises on donkeys. Recognizing the injuries and their cause can be very helpful to suggest methods to avoid these injuries in future (56).

The results of this study suggest that the use of a whip was very rare and this may be due to the availability of a rope fitted to the mouth to guide the movement of cart-donkeys. Donkey owners with higher average household incomes of $200–250 per month exhibited an optimum body condition of the donkey (OR= 4.25, Cl = 2.75–20.71) due to adequate nutrition (OR = 1.97, Cl = 0.95–4.0) and indicates daily working hours exceeding six. This indicated an improvement in overall healthcare management and the welfare of donkeys. However, the real situation is quite different.

This study also demonstrated a high prevalence of hyperlipaemia and lameness among donkey's population and represented a significant welfare concern. Health problems such as fatty liver diseases, gastric ulceration, hyperlipidemia and lameness are very widespread among donkeys (30, 57). Several studies have also reported other health problems in donkeys such as a history of metabolic syndrome (58), metabolic disorders (59) and respiratory tract disorders (60). We have not observed these health problems among donkeys in our study. Among these health-related problems, the most common disorder is hyperlipemia in donkeys, with mortality rates of up to 60–80% (61). The prevalence of hyperlipaemia in the present study was lower than those reported elsewhere in cart donkeys (30). The frequency of preventive healthcare interventions, such as vaccination, cleaning of eyes and dental care for donkeys were lower in the current study, which was similar to the frequency reported for working donkeys in most developing countries such as Ethiopia and Pakistan (20). Dental disorders in donkeys can promote incisor diastemata, sharp teeth and increased focal growth (62). Most of the respondents in our investigation were not regularly cleaning the eyes of their donkeys. This observation is comparable with a previously published report which suggests that the cart-donkeys with incorrect blinkers may have a higher risk of developing eye problems such as inflammation and discharge of ocular and traumatic wounds (63).

Donkeys are at greater risk of obesity compared to horses in developed countries (64). Recent studies have shown that obese donkeys have higher insulin values which may increase the risk of a donkey to develop laminitis (65). However, the situation is quite different in developing countries including Pakistan, where donkeys have more than 6 h as their working hours. Our research revealed that most of donkey were of normal weight. Some donkeys were also noted underweight due to an inadequate nutrition.

It was a matter of great concern that none of the donkeys involved in our study were vaccinated against any of the diseases, which clearly indicates the poor condition of the preventive management and healthcare of the donkeys in Balochistan. There are few commercial vaccines for horses in Balochistan, but this facility is not available to donkeys.

There were several constraints in this research such as the lack of reliable and up-to-date data on donkeys. There are challenges in obtaining accurate statistics on the donkey population and estimating their economic value for a country (66). The donkey owners work 6 days a week and have a holiday either on Friday or Sunday. As donkeys are used for a variety of activities, therefore, their management is different from other animals.

## Conclusions

Donkeys have become more popular among small farmers than horses and mules. In Pakistan, there is little concern about the healthcare management and welfare of donkeys. This study revealed that working donkeys face health and management problems that hinder their efficient use. The health problems include the diseases of eye, teeth, skin and lameness are very common among donkeys. The control and medical treatment of these diseases is poorly managed. The management problems are housing, feeding, grooming and hygiene of donkeys. Unfortunately, donkeys are not being given appropriate attention and preference in the livestock programs by policymakers. Therefore, there is a need to develop specific management methods which will allow donkeys to fully maximize their natural survival benefits i.e., life span. Veterinarians and animal welfare organizations can play the main role in creating awareness and in creating awareness and distributing the guidelines materials in the local language for the protection of donkeys, but it is absent. For the sustainable growth of the donkey population, we must provide them with good healthcare management and welfare. There is a need to promote the donkey vaccination program to increase the individual immunity of donkeys and reduce the incidence and risk of diseases.

## Data availability statement

The original contributions presented in the study are included in the article/Supplementary material, further inquiries can be directed to the corresponding author/s.

## Ethics statement

The animal study was reviewed and approved by Research Ethics Committee University of Balochistan Quetta. Written informed consent was obtained from the individual(s) for the publication of any potentially identifiable images or data included in this article.

## Author contributions

Conceptualization: AAl. Investigation: KK, AAk, and AS. Methodology: KK and AAk. Supervision: AAl and MS. Resources: S, MN, JA, and ZR. Writing-review and original draft: KK, AAl, and MS. All authors contributed to the article and approved the submitted version.

## Conflict of interest

The authors declare that the research was conducted in the absence of any commercial or financial relationships that could be construed as a potential conflict of interest.

## Publisher's note

All claims expressed in this article are solely those of the authors and do not necessarily represent those of their affiliated organizations, or those of the publisher, the editors and the reviewers. Any product that may be evaluated in this article, or claim that may be made by its manufacturer, is not guaranteed or endorsed by the publisher.
