# Supplementary material for: Participatory appraisal for healthcare and welfare management strategies of donkeys (Equus ascinus) in Balochistan, Pakistan
Source: Front Vet Sci. 2022 Sep 2;9:1005079. doi: 10.3389/fvets.2022.1005079 (PMC9478909; doi:10.3389/fvets.2022.1005079)

Supplementary Material S1. The figures show the estimated highest donkey population in the top 13 countries in 2018. The intensity of shading gives an approximate visual indication of the numbers of donkeys in the different countries (source: http://www.fao.org/faostat/en/).

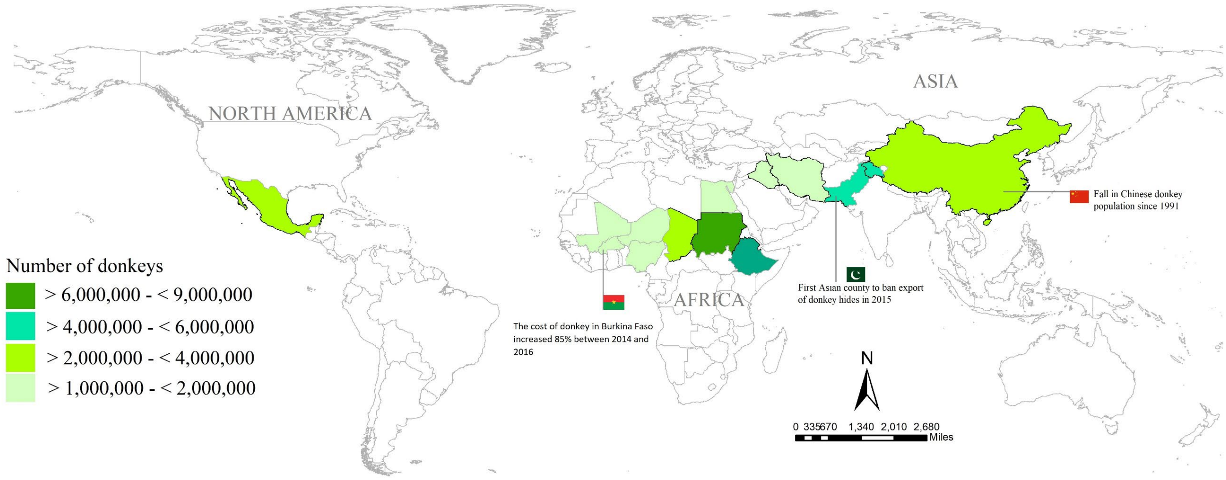

Supplement: Supplementary file 1 [file Image_1.pdf]
