# Supplementary material for: Participatory appraisal for healthcare and welfare management strategies of donkeys (Equus ascinus) in Balochistan, Pakistan
Source: Front Vet Sci. 2022 Sep 2;9:1005079. doi: 10.3389/fvets.2022.1005079 (PMC9478909; doi:10.3389/fvets.2022.1005079)
Supplement: Supplementary file 3 [file Data_Sheet_1.docx]

**Supplementary Material S3.** Questionnaire on the knowledge, attitudes and practices towards healthcare management and welfare of domestic donkeys.

**Healthcare Management and Welfare of Domestic Donkeys**


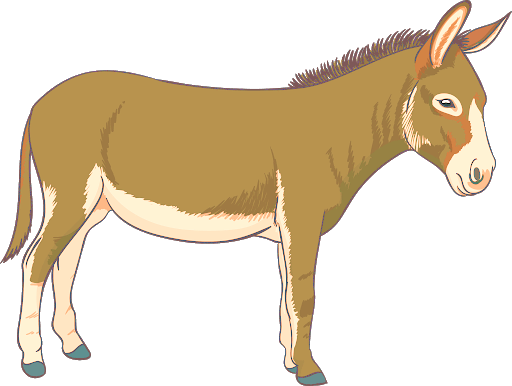


**General information**

1. Name of district: ________________
2. Name: First name ________________ Surname_________________
3. Home address: ________________
4. Subject ID: ___ ___ ___ ___
5. Date of interview (*dd/mm/yyyy*): ____/____/____
6. Time of interview (00-24): /
7. Language used for interview: ________________
8. Mobile number: ________________

| **Section I Socio-demographic based questions** | |
| --- | --- |
| A1 | Age of participant |
| A2 | What area does the donkey owner belong to? |
| A3 | What is your marital status? |
| A4 | What is the level of your education? |
| A5 | Do the donkeys belong to you or hired? |
| A6 | How long is your experience working with donkeys? |
| A7 | How many donkeys do you have? |
| A8 | Do you have any other animals besides donkeys? |
| A9 | What is your main source of income? |
| A10 | What is your monthly income? |
| A11 | Do you also provide farrier service to donkey? |
| A12 | Are you native of Pakistan? |
| A13 | How do you use donkeys in everyday life? |
| A14 | What is the number of people in your household? |

| **Section II Knowledge based questions** | |
| --- | --- |
| B1 | Are you aware of zoonotic diseases? |
| B2 | Do you know about tick-borne, lice and mites’ diseases in donkeys? |
| B3 | Do you have any shelter for donkeys? |
| B4 | Have you ever noticed a loss of weight in donkey due to ticks? |
| B5 | Do you provide first aid if the donkey is sick? |
| B6 | Is the sick donkey given proper rest? |
| B7 | Does the government conduct surveys to assess health of donkey? |
| B8 | Are you aware of medical care centres for donkey in your locality? |
| B9 | Does the government provide full support in case of illness of donkey? |
| B10 | Do you have any trouble to explain donkey health condition to a veterinary officer? |
| B11 | Is the donkey vaccinated against influenza and tetanus? |
| B12 | Diseases or symptoms usually noticed in donkey? |
| B13 | What is the cost of a single donkey? |
| B14 | Has the government tagged your animal in the ear? |

| **Section III Attitude based questions** | |
| --- | --- |
| C1 | Is the donkey kept with other animals? |
| C2 | When in contact with other livestock do you adopt a personal protection strategy? |
| C3 | Do you participate in free donkey health training? |
| C4 | How many times a day do you feed your donkey? |
| C5 | What fodder is used for donkey? |
| C6 | How many times you clean your donkey? |
| C7 | How many times serve water to donkey? |
| C8 | Do you server clean drinking water? |
| C9 | In case of ectoparasite on the body of donkey, do you consult a veterinarian? |
| C10 | How many times you whipped your donkey in the single day? |
| C11 | Does the government provide microchips for donkeys? |
| C12 | Does the donkey have a comfortable sleeping bed? |
| C13 | Have you travelled outside of your district in the last six months? |
| C14 | Do you rent a donkey cart? |
| C15 | Who takes care of your animal when you are sick? |

| **Section IV Practice related questions** | |
| --- | --- |
| D1 | Do you use chemicals (acaricides) to cure tick infestation? |
| D2 | For many days you use your donkey for farriery services? |
| D3 | What is the main reason for farriery services? |
| D4 | Do you sell the donkey for slaughtering? |
| D5 | Have you vaccinated your donkey? |
| D6 | Do you receive any financial support? |
| D7 | What kind of weight is carried by the donkey? |
| D8 | How much weight does a donkey usually carry in a day? |
| D9 | What is the during of working hours of donkeys? |
| D10 | Do you know the maximum standard limit of working hours for a donkey? |
| D11 | What is the standard weight carried by a donkey? |
| D12 | How many days in week do you work? |
| D13 | Do you take a break in a weekend? |
